# Dimethylaminoparthenolide and gemcitabine: a survival study using a genetically engineered mouse model of pancreatic cancer

**DOI:** 10.1186/1471-2407-13-194

**Published:** 2013-04-17

**Authors:** Michele T Yip-Schneider, Huangbing Wu, Keith Stantz, Narasimhan Agaram, Peter A Crooks, C Max Schmidt

**Affiliations:** 1 Department of Surgery, Indiana University School of Medicine, 980 W. Walnut St., Building R3, Rm. 541C, Indianapolis, IN 46202, USA; 2 Department of Radiology, Indiana University School of Medicine, Indianapolis, IN, USA; 3 Department of Pathology, Indiana University School of Medicine, Indianapolis, IN, USA; 4 Department of Biochemistry/Molecular Biology, Indiana University School of Medicine, Indianapolis, IN, USA; 5 Department of Walther Oncology Center, Indiana University School of Medicine, Indianapolis, IN, USA; 6 Indiana University Cancer Center, Indianapolis, IN, USA; 7 Richard L. Roudebush VA Medical Center, Indianapolis, IN, USA; 8 Department of Pharmaceutical Sciences, College of Pharmacy, University of Arkansas for Medical Sciences, Little Rock, AR, USA

**Keywords:** Pancreatic cancer, Therapy, Chemoprevention, Parthenolide, Gemcitabine, NF-κB

## Abstract

**Background:**

Pancreatic cancer remains one of the deadliest cancers due to lack of early detection and absence of effective treatments. Gemcitabine, the current standard-of-care chemotherapy for pancreatic cancer, has limited clinical benefit. Treatment of pancreatic cancer cells with gemcitabine has been shown to induce the activity of the transcription factor nuclear factor-kappaB (NF-κB) which regulates the expression of genes involved in the inflammatory response and tumorigenesis. It has therefore been proposed that gemcitabine-induced NF-κB activation may result in chemoresistance. We hypothesize that NF-κB suppression by the novel inhibitor dimethylaminoparthenolide (DMAPT) may enhance the effect of gemcitabine in pancreatic cancer.

**Methods:**

The efficacy of DMAPT and gemcitabine was evaluated in a chemoprevention trial using the mutant Kras and p53-expressing *LSL-Kras*^*G12D/+*^*; LSL-Trp53*^*R172H*^*; Pdx-1-Cre* mouse model of pancreatic cancer. Mice were randomized to treatment groups (placebo, DMAPT [40 mg/kg/day], gemcitabine [50 mg/kg twice weekly], and the combination DMAPT/gemcitabine). Treatment was continued until mice showed signs of ill health at which time they were sacrificed. Plasma cytokine levels were determined using a Bio-Plex immunoassay. Statistical tests used included log-rank test, ANOVA with Dunnett’s post-test, Student’s *t*-test, and Fisher exact test.

**Results:**

Gemcitabine or the combination DMAPT/gemcitabine significantly increased median survival and decreased the incidence and multiplicity of pancreatic adenocarcinomas. The DMAPT/gemcitabine combination also significantly decreased tumor size and the incidence of metastasis to the liver. No significant differences in the percentages of normal pancreatic ducts or premalignant pancreatic lesions were observed between the treatment groups. Pancreata in which no tumors formed were analyzed to determine the extent of pre-neoplasia; mostly normal ducts or low grade pancreatic lesions were observed, suggesting prevention of higher grade lesions in these animals. While gemcitabine treatment increased the levels of the inflammatory cytokines interleukin 1α (IL-1α), IL-1β, and IL-17 in mouse plasma, DMAPT and DMAPT/gemcitabine reduced the levels of the inflammatory cytokines IL-12p40, monocyte chemotactic protein-1 (MCP-1), macrophage inflammatory protein-1 beta (MIP-1β), eotaxin, and tumor necrosis factor-alpha (TNF-α), all of which are NF-κB target genes.

**Conclusion:**

In summary, these findings provide preclinical evidence supporting further evaluation of agents such as DMAPT and gemcitabine for the prevention and treatment of pancreatic cancer.

## Background

Pancreatic adenocarcinoma is the fourth leading cause of cancer-related deaths in the United States, with mortality nearly equal to incidence. Less than 5% of patients survive five years from the time of diagnosis, and the median survival time is less than 6 months. This year alone, pancreatic cancer will result in approximately 40,000 deaths in the United States [1]. At the time of diagnosis, surgical resection is unfortunately not an option for many patients due to the advanced stage of disease and distant metastases. In addition, current chemotherapeutic strategies are largely ineffective because of either innate or acquired chemoresistance. Gemcitabine (2’,2’-difluorodeoxycytidine) is the drug of choice for the primary treatment of unresectable pancreatic cancer and adjuvant treatment following resection of pancreatic cancer, but measurable responses are not observed in the majority of patients [2,3]. Fortunately, gemcitabine, compared to other chemotherapeutic agents, is relatively non-toxic [4]; however, its use as chemoprevention in patients with known precancerous lesions has not been explored. Other chemotherapeutic agents, e.g., paclitaxel, have recently been used in patients with precancerous pancreatic lesions with some evidence of regression [5].

To aid in the search for effective prevention and intervention strategies, clinically relevant animal models are needed and have recently been developed [6]. For example, a genetically engineered mouse model of pancreatic cancer with targeted expression of mutant *KRAS*^*G12D*^ and *Trp53*^*R172H*^ genes has been shown to recapitulate human pancreatic neoplasia, from premalignant lesions to invasive cancer and metastasis [7]. The *LSL-Kras*^*G12D/+*^*; LSL-Trp53*^*R172H*^*; Pdx-1-Cre* mice are a developmental model of pancreatic cancer in which adenocarcinoma form *de novo* with close to 100% penetrance. In this mouse model, the Lox-Stop-Lox (LSL) sequence upstream of oncogenic *KRAS* and mutant *Trp53* inhibits transcription and translation. Expression of Cre recombinase from the pancreatic-specific promoter Pdx-1, excision of the “Stop” sequences, and subsequent Cre-mediated recombination allow endogenous expression of the mutant Kras and p53 in progenitor cells of the mouse pancreas. Another advantage of this model is that the natural microenvironment of the pancreas is maintained. Thus, preclinical data from these types of animal models may be more predictive of human clinical outcomes.

Due to its critical role in inflammation and multiple tumorigenic processes, the transcription factor nuclear factor-kappaB (NF-κB) is a therapeutic target of interest for pancreatic cancer [8,9]. In addition, the p65 subunit of NF-κB, RelA, is constitutively active in human pancreatic adenocarcinoma tissue and in pancreatic tumor cell lines [10]. It was recently demonstrated in a genetically engineered mouse model that constitutive NF-κB activation, by Kras through AP-1-induced overexpression of interleukin-1α (IL-1α), is required for the development of pancreatic cancer [11]. These findings implicate NF-κB in the development and progression of pancreatic cancer. Furthermore, experimental evidence suggests that NF-κB may also be a suitable target for chemoprevention [12,13]. We have previously examined the anti-cancer activity of dimethylaminoparthenolide (DMAPT), which is a novel and orally bioavailable analog of parthenolide, a sesquiterpene lactone isolated from the medicinal herb feverfew (*Tanacetum parthenium*) [14]. In both xenograft and carcinogen-induced animal models of pancreatic cancer, DMAPT inhibits the activity of NF-κB and shows therapeutic promise in combination with the anti-inflammatory agents sulindac or celecoxib *in vivo*[15,16].

We and others have also reported that the chemotherapeutic agent gemcitabine induces NF-κB activity in pancreatic cancer cells *in vitro*, suggesting that NF-κB activation may play a role in chemoresistance to gemcitabine [9,17-20]. A viable strategy for improving the therapeutic response to gemcitabine may therefore involve suppression of the NF-κB pathway. In support, we recently demonstrated that DMAPT not only inhibits gemcitabine-induced NF-κB activation but also sensitizes pancreatic cancer cells to the anti-proliferative effects of gemcitabine *in vitro*, indicating that the level of NF-κB activity modulates the gemcitabine response [21]. Furthermore, in a heterotopic xenograft model, gemcitabine exposure activates NF-κB within established pancreatic tumors, suggesting that NF-κB suppression may also improve the anti-tumor effects of gemcitabine *in vivo*[21]. Most recently, we found that DMAPT and/or sulindac in combination with gemcitabine therapy can delay or prevent progression of premalignant pancreatic lesions in the less aggressive *LSL-Kras*^*G12D/+*^*; Pdx-1-Cre* mouse model of pancreatic cancer [22]. Due to the low incidence of pancreatic tumors in the *LSL-Kras*^*G12D/+*^*; Pdx-1-Cre* mouse model, the clinical relevance of this delay on pancreatic tumor formation or metastasis could not be determined. Thus, the chemopreventative efficacy of the most effective combination DMAPT/gemcitabine was further evaluated in this survival study using the *LSL-Kras*^*G12D/+*^*; LSL-Trp53*^*R172H*^*; Pdx-1-Cre* mouse model, which is characterized by near 100% incidence of pancreatic adenocarcinoma development.

## Methods

### Compounds

Gemcitabine (GEMZAR^®^) was obtained from Eli Lilly (Indianapolis, IN). DMAPT [14] was synthesized by reaction of parthenolide (Sigma-Aldrich, St. Louis, MO) with dimethylamine (Sigma-Aldrich, St. Louis, MO) and isolated as the fumarate salt.

### LSL-Kras^G12D/+^; LSL-Trp53^R172H^; Pdx-1-Cre mouse model

This study was performed in compliance with federal Institutional Animal Care and Use Committee guidelines. Male *LSL-Kras*^*G12D/+*^*; Pdx-1-Cre* mice (breeders kindly provided by Dr. Andrew Lowy, University of California, San Diego [23]) were crossed with female *p53 LSL*^*R172H*^ (NCI-Frederick) mice to generate *LSL-Kras*^*G12D/+*^*; LSL-Trp53*^*R172H*^*; Pdx-1-Cre* mice. At 1 month of age, mice were genotyped by PCR analysis of tail genomic DNA. For Kras^G12D^, primers were as follows resulting in amplification products of 500 bp (wild-type) and 550 bp (mutant allele):

5^′^ wild type: GTCGACAAGCTCATGCGGG;

5^′^ mutant (LSL element): CCATGGCTTGAGTAAGTCTGC

3^′^ universal: CGCAGACTGTAGAGCAGCG

For Cre, the primers were as follows to generate a 475 bp amplification product:

5^′^: AGATGTTCGCGATTATCTTC

3^′^: AGCTACACCAGAGACGG

For p53^R172H^, primers were as follows generating amplification products of 166 bp (wild-type) and 270 bp (LSL element):

5^′^ mutant (LSL element): AGCTAGCCACCATGGCTTGAGTAAGTCTGC

5^′^ wild-type: TTACACATCCAGCCTCTGTGG

3^′^ universal: CTTGGAGACATAGCCACACTG

This breeding scheme resulted in ~12% positive mice which were eligible for rolling enrollment in the study.

At 1 month of age, *LSL-Kras*^*G12D/+*^*; LSL-Trp53*^*R172H*^*; Pdx-1-Cre* mice were randomized into treatment groups (placebo, DMAPT, gemcitabine, DMAPT/gemcitabine). Placebo (vehicle = hydroxylpropyl methylcellulose, 0.2% Tween 80 [HPMT]) and DMAPT (40 mg/kg body weight in HPMT) were administered by oral gastric lavage once daily. Gemcitabine (50 mg/kg body weight in PBS) was administered by intraperitoneal injection twice weekly. Mouse weight was monitored weekly. Treatment was continued until mice showed signs of lethargy, abdominal distension or weight loss at which time they were sacrificed. Successful excision-recombination events were confirmed in the pancreata of mice by detecting the presence of a single LoxP site [24].

Upon necropsy, the presence and size of gross pancreatic tumors and metastases were noted. The presence of multiple tumors was determined both by gross examination and palpation of the pancreas since the boundaries between multiple large tumors can be difficult to delineate in hematoxylin and eosin (H&E)-stained specimens. In these cases, gross tumor dimensions were used for analysis. For the smaller tumors, identification of pancreatic ductal adenocarcinoma, as well as their dimensions, was confirmed upon review of ten consecutive H&E-stained sections per pancreas (100 μm apart) by a pathologist blinded to the experimental groups. Tumor volume was calculated using a modified ellipsoidal formula, 1/2(length × width^2^). Liver, kidney, and lung were also examined for signs of drug toxicity. Pancreatic, liver and lung tissue pieces (3 mm) were frozen in liquid nitrogen and stored at −80°C for analysis; the remaining tissues were fixed in 10% formalin (Sigma, St. Louis, MO) and paraffin-embedded for H&E staining and immunohistochemistry. Serial liver and lung sections (10–15 sections each, 100 μm apart) were examined for metastases. Blood (~ 1 ml) was obtained by cardiac puncture, mixed with 25 μl anticoagulant (EDTA [2 g]/NaCl [0.8 g] in 100 ml water, pH 7.4) and centrifuged (2800 rpm, 15 minutes, 4°C). Plasma aliquots were frozen at −80°C.

### Luciferase-expressing p53^f/f^; LSL-Kras^G12D^;luc^l/l^; Pdx-1-Cre mouse model

*Floxed-p53/LSL-Kras*^*G12D*^*/floxed-STOP-luciferase* male mice (designated *p53*^*f/f*^*; LSL-Kras*^*G12D*^*;luc*^*l/l*^, breeders kindly provided by Dr. Robert Bigsby, Indiana University [25]) were crossed with *Pdx-1-Cre* mice to generate *p53*^*f/f*^*; LSL-Kras*^*G12D*^*;luc*^*l/l*^*; Pdx-1-Cre* mice, in which p53 is deleted and mutant Kras and luciferase are expressed. After genotyping, mice were randomized into single agent treatment groups (placebo, DMAPT and gemcitabine) at two months of age as described above. Following injection with D-luciferin (60 mg/kg, in 0.3 mL PBS) into the intraperitoneal cavity, imaging was performed using the NightOWL optical Imager (LB981, Berthold) to detect luciferase expression within the pancreas.

### Mouse PanIN (mPanIN) analysis

One section per pancreas, with maximal exposure (greater than 75%) of the pancreas (5 μm) was cut, stained with hematoxylin and eosin (H&E), and examined microscopically for lesions. Sections from all animals in each treatment group were analyzed. mPanINs were counted in a blinded manner according to previously established criteria [26,27]. The highest grade lesion in the individual pancreatic lobules within the entire pancreas from each animal was identified for quantification. The percent normal ducts, mPanIN-1, mPanIN-2 and mPanIN-3 lesions was determined relative to the total number of lesions counted per pancreas.

### Immunohistochemistry and staining

Immunohistochemistry was performed with primary antibodies NF-κB/p65 (1:400, Lab Vision Corporation, Fremont, CA), phospho-ERK (1:500, Cell Signaling, Danvers, MA), Ki67 (1:50, Dako North America, Carpinteria, CA) and CD31 (1:20, Dianova, Hamburg, Germany). Briefly, slides were deparaffinized and hydrated in running water. Slides were placed in Antigen Retrieval Citrate Buffer pH 6.0 (Dako North America) in a pressure cooker for 15 minutes before placing in 3% H_2_O_2_ for 10 min. All slides were placed in Protein Block (Dako North America) for 15 min, incubated with appropriate primary and secondary antibodies, and then counterstained. Percent NF-κB and Ki67 staining was quantified by counting the number of positively staining tumor cells in two fields with the highest density of staining per pancreas and expressed relative to the total number of cells within the field. Intratumoral CD31 staining was quantified using ImageScope software and the positive pixel count algorithm (Aperio Technologies, Vista, CA).

Masson’s Trichrome was performed using Sigma-Aldrich Accustain Trichrome Stains (Masson, kit No. HT-15) and quantified using ImageScope software.

### Cytokine analysis

Mouse plasma obtained at the time of sacrifice was analyzed using the Bio-Plex Pro™ mouse cytokine 23-plex immunoassay (Biorad, Hercules, CA) and the Bio-Plex 200 System, as recommended by the manufacturer. Samples were diluted 1:4 with sample diluent supplied in the Bio-Plex kit prior to analysis. Analyte values that were out of range or with a low bead count (< 50) were excluded from analysis.

### Statistical Analysis

Median survival was determined by the Kaplan-Meier method and analyzed by the log-rank test. Comparisons between placebo and treatment groups were analyzed by ANOVA with Dunnett’s post-test or Student’s *t*-test (Prism 5.0 software, Graphpad, San Diego, CA). For incidence, Fisher exact test was performed. P < 0.05 was considered significant (two-tail, 95% confidence interval).

## Results

### Treatment of LSL-Kras^G12D/+^; LSL-Trp53^R172H^; Pdx-1-Cre mice

To test the chemopreventative efficacy of DMAPT and/or gemcitabine, *LSL-Kras*^*G12D/+*^*; LSL-Trp53*^*R172H*^*; Pdx-1-Cre* mice were randomized into one of the following treatment groups at 1 month of age: placebo (n = 14), DMAPT (40 mg/kg/day, n = 15), gemcitabine (50 mg/kg/dose, n = 14), or DMAPT/gemcitabine (n = 15). The dose of DMAPT chosen for this study was the same as that previously shown by our laboratory to inhibit NF-κB in other animal models of pancreatic cancer [15,16,22]. DMAPT is currently being evaluated in clinical trials; however doses of 50-100 mg/kg have been employed *in vivo* in canine and mouse models to inhibit NF-κB, without any evidence of toxicity [28]. The gemcitabine dose of 50 mg/kg twice weekly is equivalent to a human dose of 300 mg/m^2^ using a body surface area (BSA) normalization method; this is relatively low compared to the standard human dosing regimen of 1000 mg/m^2^ given once per week for 7 weeks [29-31].

### Gemcitabine or the combination DMAPT/gemcitabine prolong survival

**Figure 1:**
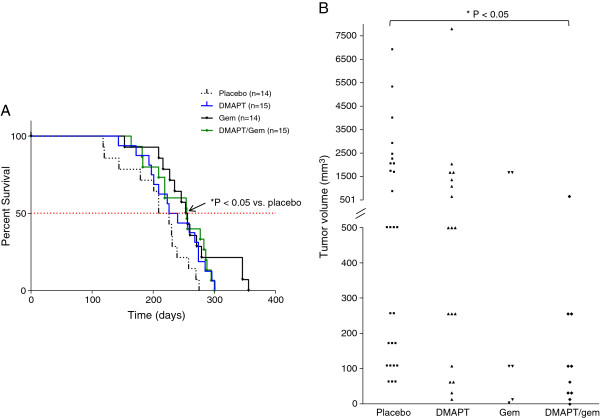
**The effect of treatment on median survival and tumor volume in *LSL-Kras***^***G12D/+***^***; LSL-Trp53***^***R172H***^***; Pdx-1-Cre *mice. A**) Median survival for each treatment group is shown in the Kaplan-Meier survival curve (placebo = 217.5 days; DMAPT = 233 days; Gem = 254.5 days; DMAPT/Gem = 255 days). * P < 0.05 for gemcitabine and DMAPT/gemcitabine vs. placebo by log-rank test. **B**) The tumor volume of individual pancreatic tumors within each treatment group is shown in the scatter plot. Note the difference in scale. Gem, gemcitabine. * P < 0.05 vs. placebo.

Treatment of the *LSL-Kras*^*G12D/+*^*; LSL-Trp53*^*R172H*^*; Pdx-1-Cre* mice with either gemcitabine or the combination DMAPT/gemcitabine significantly increased the median survival time by more than 30 days compared to the placebo group (254.5 [P = 0.015] or 255 days [P = 0.018] vs. 217.5 days, respectively) (Figure 1A). The median survival for the DMAPT-treated mice (233 days), although longer, was not significantly different from the placebo group. No significant weight loss during the course of the study or other gross evidence of drug toxicity was noted in the treatment groups.

**Figure 2:**
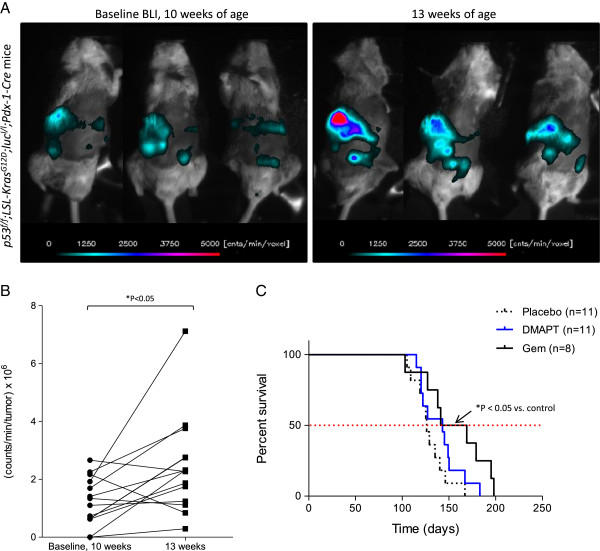
**Luciferase-expressing *p53***^***f/f***^***; LSL-Kras***^***G12D***^***;luc***^***l/l***^***; Pdx-1-Cre *mice. A**) At 10 and 13 weeks of age, *p53*^*f/f*^*; LSL-Kras*^*G12D*^*;luc*^*l/l*^*; Pdx-1-Cre* mice were injected with D-luciferin (60 mg/kg; i.p.) and imaged to detect bioluminescence as shown for three representative mice. At each time point, a sequence of 15 images (2 minutes exposure time/image) was acquired. The image with the peak bioluminescence was used to assess relative change in photon fluence rate, e.g., counts/min in each voxel, using a lower threshold of 150 counts/min/sec (> 10 times background noise). The optical geometry was identical for all imaging sessions. **B**) Total photon flux (counts/min/tumor x 10^6^) at 10 and 13 weeks of age (n = 14 mice). * P < 0.05 by two-tailed paired *t*-test. **C**) Median survival for each treatment group is shown in the Kaplan-Meier survival curve (placebo = 126 days; DMAPT = 143 days; Gem = 155 days). * P < 0.05 for gemcitabine vs. placebo by log-rank test.

A pilot study was also performed with a related model suitable for imaging, *p53*^*f/f*^*; LSL-Kras*^*G12D*^*;luc*^*l/l*^*; Pdx-1-Cre* mice, in which p53 is deleted and mutant Kras and luciferase are expressed in the pancreas. The *p53*^*f/f*^*; LSL-Kras*^*G12D*^*;luc*^*l/l*^*; Pdx-1-Cre* mice were imaged at 10 weeks of age to monitor luciferase expression and detect bioluminescence (Figure 2A). All mice expressed luciferase as shown in the three representative mice, confirming the functional expression of Cre recombinase in the pancreas by 10 weeks of age; bioluminescence was also detected at 6 weeks of age, the earliest timepoint at which the animals were imaged. Three weeks after the initial imaging, the same mice were re-imaged; increased bioluminescence and therefore luciferase expression were detected, that should reflect a corresponding increase in mutant Kras expression and p53 deletion in the pancreas at 13 weeks of age. Total photon flux significantly increased between 10 and 13 weeks for the majority of the mice (Figure 2B). Using this model to test the single agents, gemcitabine was administered at 8 weeks of age and significantly increased median survival by approximately 30 days compared to placebo (155 days vs. 126 days [P = 0.02]), thus confirming gemcitabine’s beneficial effect (Figure 2C). DMAPT did not significantly extend survival (143 days [P = 0.14]).

### Effect of treatment on pancreatic tumor incidence, size, and metastasis

The presence of pancreatic tumors and metastasis in the *LSL-Kras*^*G12D/+*^*; LSL-Trp53*^*R172H*^*; Pdx-1-Cre* mice was noted upon necropsy and subsequently confirmed by histology. Pancreatic adenocarcinomas were detected in 100% of the placebo-treated mice. Tumor incidence was decreased by treatment with DMAPT (73%) and significantly by gemcitabine (43%) as well as the combination (60%) (Table 1). To confirm that the lack of tumor formation was not due to the absence of Cre-mediated recombination in the pancreas, PCR was performed demonstrating successful recombination (Additional file 1: Figure S1). Almost all of the mice without tumors that died were older (> 200 days old) and/or had other large primary tumors (lung, lymphoma, liver). Histological organ examination did not show evidence of drug toxicity.

**Table 1:** Pancreatic tumor incidence, multiplicity and metastasis

**Treatment**	**Incidence (%)**	**Incidence, T ≥ 500 mm**^**3 **^**(%)**	**Multiplicity**	**Metastasis (%)**	**Liver met (%)**	**Lung met (%)**
Placebo	14/14 (100%)	11/14 (78%)	27/14 = 1.9	7/14 (50%)	6/14 (43%)	3/14 (21%)
DMAPT	11/15 (73%)	8/11 (73%)	18/15 = 1.2	5/11 (45%)	2/11 (18%)	5/11 (45%)
Gemcitabine	6/14 (43%)*	2/6 (33%)	6/14 = 0.4*	3/6 (50%)	3/6 (50%)	1/6 (17%)
DMAPT/gemcitabine	9/15 (60%)*	1/9 (11%)*	10/15 = 0.7*	2/9 (22%)	0/9 (0%)*^/^**	2/9 (22%)

Incidence = number of mice with tumors/total number of mice (n).

Incidence, T ≥ 500 mm^3^ = number of mice with tumor volume ≥ 500 mm^3^/number of tumor-bearing mice.

Multiplicity = number of tumors/total number of mice (n).

Metastasis = number of mice with metastases/number of tumor-bearing mice.

Liver Met = number of mice with liver metastases/number of tumor-bearing mice.

Lung Met = number of mice with lung metastases/number of tumor-bearing mice.

*P < 0.05 compared to placebo.

** P < 0.05 compared to gemcitabine.

The incidence of large pancreatic adenocarcinomas greater than or equal to 500 mm^3^ was similar in the placebo (78%) and DMAPT (73%) groups but was decreased by treatment with gemcitabine (33%) and significantly with the combination (11%) (Table 1). Furthermore, pancreatic tumor volume was significantly decreased by treatment with the combination DMAPT/gemcitabine compared to placebo (153 +/− 63.9 mm^3^ vs. 1329 +/− 338.2 mm^3^, respectively) (Figure 1B). Although tumor volume was also reduced by gemcitabine (601 +/− 343.9 mm^3^), this did not reach significance due to the variability in response (Figure 1B).

Tumor multiplicity was also significantly reduced by treatment with gemcitabine or DMAPT/gemcitabine compared to placebo (0.4 or 0.7 vs. 1.9 tumors/mouse, respectively) (Table 1). Although the incidence of metastasis was the lowest in the DMAPT/gemcitabine treated mice, the decrease was not significant; however, the incidence of liver metastasis in mice bearing primary pancreatic tumors was significantly reduced from ~50% in the placebo and gemcitabine groups to 0% in the DMAPT/gemcitabine treated mice (Table 1). Interestingly, while metastasis occurred to the liver and lung within the placebo, DMAPT, and gemcitabine groups, 100% of the mice in the DMAPT/gemcitabine group formed metastases in the lung only (Table 1); statistical significance cannot be determined due to the low numbers. Taken together, these results demonstrate that the combination of DMAPT and gemcitabine is more effective than the single agents, significantly decreasing pancreatic tumor size as well as the incidence of liver metastasis.

### Histological analysis of pancreatic lesions

**Figure 3:**
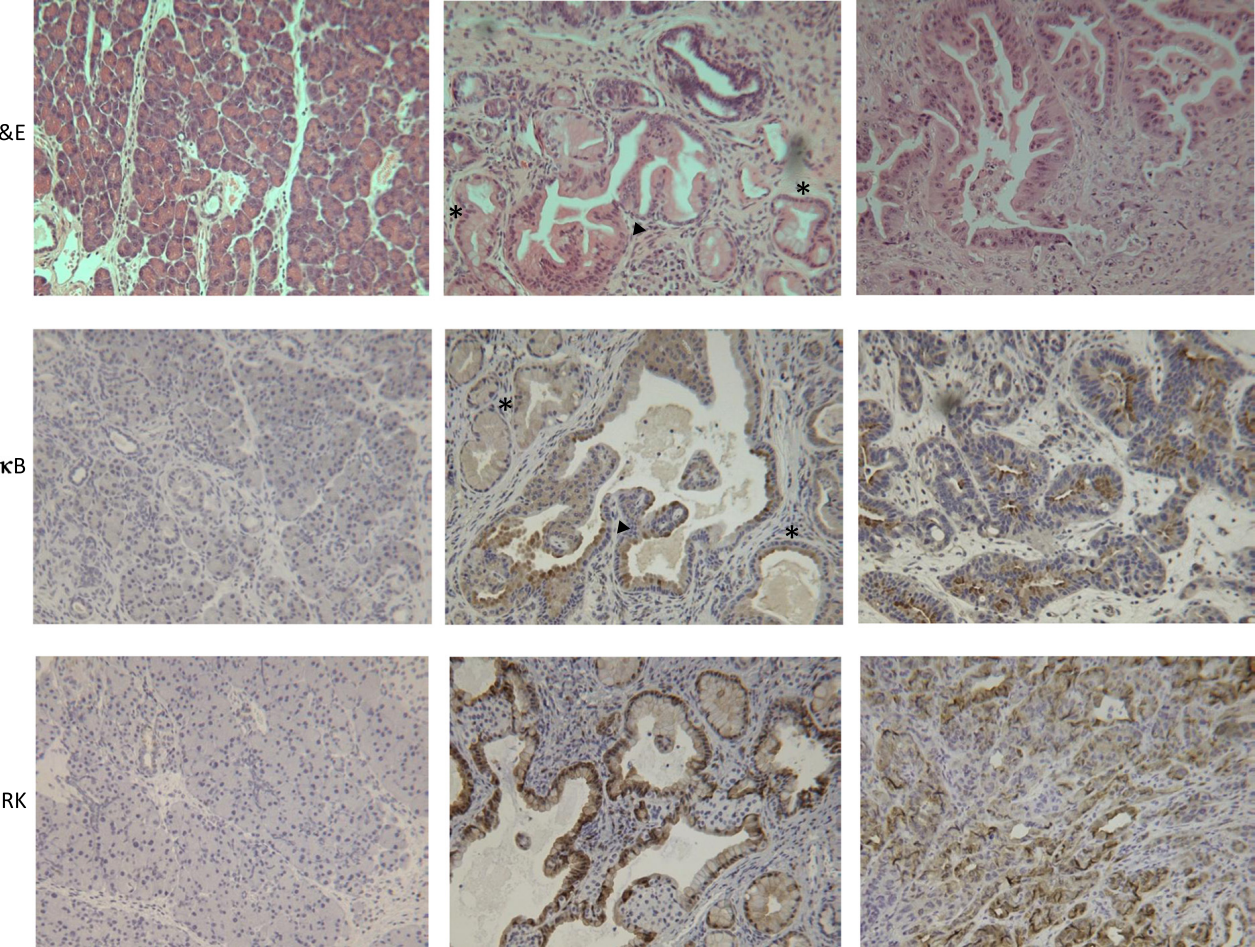
**Pancreas histology and expression of NF-κB and P-ERK. A**) H&E. Representative sections of normal adjacent pancreas upon sacrifice at day 219 [d219]), mPanIN-1 (asterisk) and −2 (black arrowhead) at d271, and pancreatic ductal adenocarcinoma at d258 are shown (200X magnification). **B**) NF-κB. Pancreatic tissue sections were immunostained with a NF-κB specific antibody. Representative images of normal adjacent pancreas at d231, mPanIN-1 (asterisk) and −2 (black arrowhead) at d226, and adenocarcinoma at d239 are shown (200X magnification). NF-κB is expressed in mPanINs and tumor cells (brown). **C**) P-ERK. Positive P-ERK staining (brown) was localized to the mPanINs (d226) and tumor cells (d295) but was absent in normal adjacent pancreas (d226) (200X magnification).

**Figure 4:**
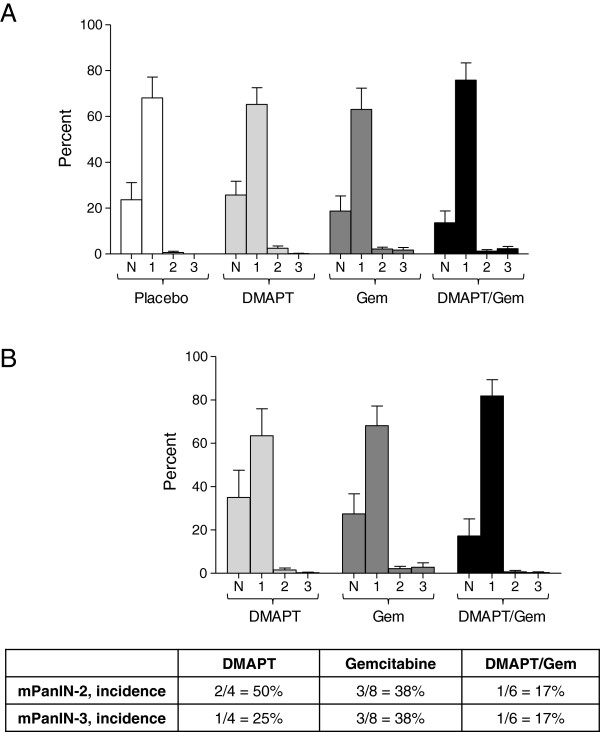
**PanIN analysis. A**) The percent normal ducts (N), mPanIN-1 (1), mPanIN-2 (2) and mPanIN-3 (3) for all pancreata within each treatment group is shown as the mean +/− SEM. **B**) The percent normal ducts and mPanIN-1, -2 and −3 for pancreata without tumors is presented as the mean +/− SEM. The incidence of mPanIN-2 and mPanIN-3 in pancreata lacking tumors is shown in the table below the graph.

Premalignant lesions, known as pancreatic intraepithelial neoplasia (PanINs), arise in the pancreas and are precursors to invasive pancreatic ductal adenocarcinoma [32]. All stages of mouse PanINs (mPanINs) and pancreatic adenocarcinoma, mirroring those observed in humans, are represented in the *LSL-Kras*^*G12D/+*^*; LSL-Trp53*^*R172H*^*; Pdx-1-Cre* mice (Figure 3A). The percentages of normal pancreatic ducts and mPanIN-1, -2 and −3 were quantified for each of the treatment groups; however, no significant differences were observed (Figure 4A). Few mPanINs were present in pancreata bearing large tumors. In addition, pancreata in which no tumors formed were separately analyzed to determine the extent of pre-neoplasia (Figure 4B); no significant differences in the % normal ducts or pancreatic lesions were observed between the three drug treatment groups. Interestingly, mostly normal ducts or low grade PanIN-1 lesions were observed in these pancreata, suggesting that not only tumor formation but also the development of higher grade pancreatic lesions is prevented in these animals. This was also confirmed by the incidence of PanIN-2 and −3 lesions in pancreata lacking tumors (Figure 4B, table), with the lowest incidence in the DMAPT/Gem treatment group (17%).

Immunohistochemistry was performed on pancreatic sections to localize the expression of NF-κB, which was found to be expressed in cells lining the mPanINs as well as in pancreatic adenocarcinoma cells (Figure 3B). The over-expression of NF-κB in non-invasive and invasive pancreatic neoplasms, but not in normal pancreatic cells, provides evidence that NF-κB is a promising target for chemoprevention and chemotherapy in this model. Immunohistochemical staining revealed that phosphorylated mitogen-activated protein kinase/extracellular signal-regulated kinase 1/2 (P-MAPK/ERK) was strongly expressed in both PanINs and tumor cells, suggesting activation of the MAPK/ERK growth pathway in the *LSL-Kras*^*G12D/+*^; *LSL-Trp53*^*R172H*^*; Pdx-1-Cre* pancreatic lesions but not in histologically normal mouse pancreas (Figure 3C).

**Figure 5:**
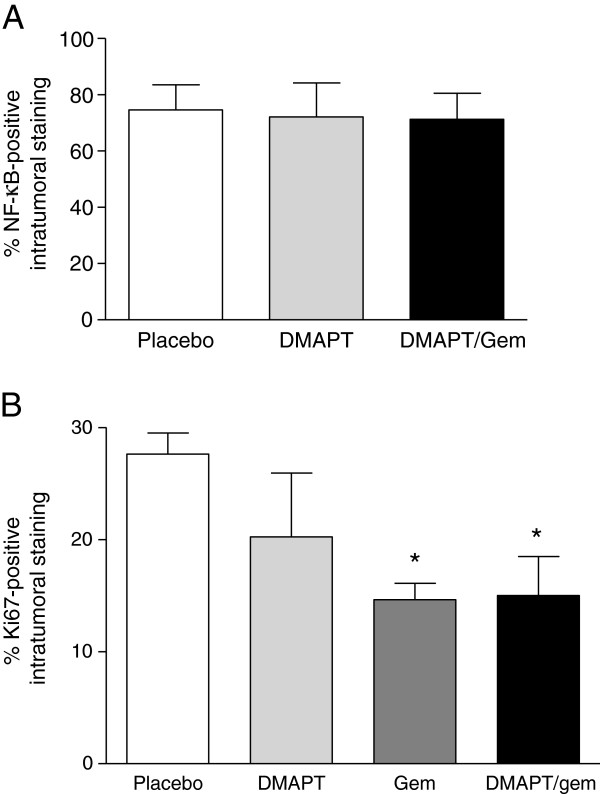
**NF-κB and Ki67 staining. A**) Percent intratumoral NF-κB staining is shown for the indicated treatment groups (n = 5-6 mice/group). **B**) Percent Ki67-positive intratumoral staining is shown (n = 4 mice/group). Results are presented as the mean +/− SEM. * P < 0.05 vs. placebo.

**Figure 6:**
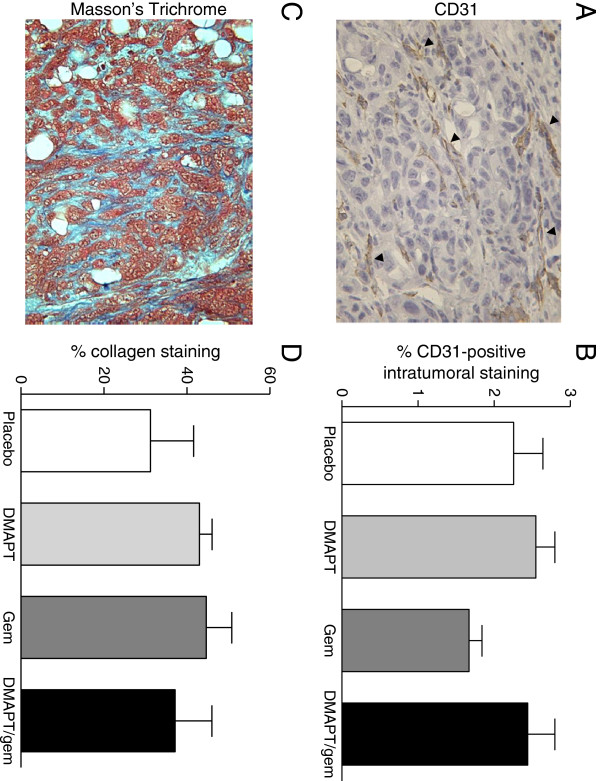
**CD31 and Masson’s Trichrome staining. A**) CD31 immunostaining. CD31-positive microvessels (brown) stained as indicated by the black arrowheads (400X magnification). **B**) Percent CD31-positive intratumoral staining within each treatment group (n = 4-5 mice/group) is shown as the mean +/− SEM. **C**) Masson’s Trichrome staining to detect the stromal component (blue) is shown (400X magnification). **D**) Percent collagen staining within each treatment group (n = 5-6 mice/group) is shown as the mean +/− SEM.

Since the animals in this study were not sacrificed at a set endpoint, optimal evaluation of target inhibition at a defined timepoint after the last drug dose was not possible. Nevertheless, intratumoral NF-κB immunohistochemical staining was quantified (Figure 5A), but no significant difference in NF-κB expression was observed between placebo and the DMAPT treatment groups. Quantification of intratumoral Ki67-positive staining showed a significant decrease in both the gemcitabine and combination groups (Figure 5B), correlating with the observed anti-tumor effects of these agents. Ki67 staining in the PanIN lesions did not differ significantly (data not shown). Pancreatic tissue sections were also stained with a CD31-specific antibody and Masson’s Trichrome to detect changes in intratumoral vasculature or stroma, respectively (Figures 6A & C). Intratumoral CD31 expression was quantified and there was no significant difference between treatment groups (Figure 6B). Similarly, there was no significant difference in the percentage of stroma/collagen as determined by quantification of Masson’s Trichrome staining (Figure 6D).

### DMAPT decreases the level of NF-κB-regulated inflammatory cytokines in mouse plasma

**Figure 7:**
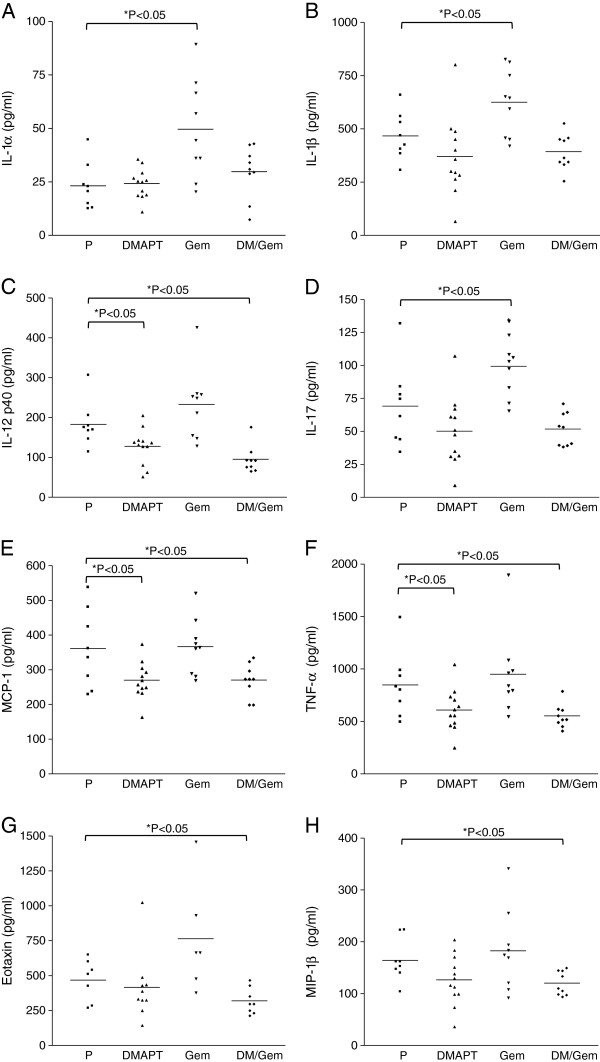
**Plasma levels of NF-κB regulated inflammatory cytokines.** Mouse plasma obtained at the time of sacrifice was analyzed using the Bio-Plex 200 system to determine the concentration of 23 different cytokines and growth factors. The levels of **A**) IL-1α, **B**) IL-1β, **C**) IL-12p40, **D**) IL-17, **E**) MCP-1, **F**) TNF-α, **G**) Eotaxin, and **H**) MIP-1β in placebo (P, n = 8), DMAPT (n = 12), gemcitabine (Gem, n = 10) and DMAPT/gemcitabine (DM/Gem, n = 9) groups are shown. The means are indicated by the horizontal lines. * P < 0.05 vs. placebo.

To identify possible indicators or mediators of drug response, mouse plasma obtained at the time of sacrifice was analyzed using the Bio-Plex 200 system that can simultaneously detect the level of 23 different cytokines and growth factors (interleukin [IL]-1α, IL-1β, IL-2, IL-3, IL-4, IL-5, IL-6, IL-9, IL-10, IL-12[p40], IL-12[p70], IL-13, IL-17, eotaxin, granulocyte-colony stimulating factor [G-CSF], granulocyte-macrophage colony stimulating factor [GM-CSF], interferon-gamma [IFN-γ], interleukin-8 homologue KC, monocyte chemotactic protein-1 [MCP-1, MCAF], macrophage inflammatory protein-1 alpha [MIP-1α], MIP-1β, RANTES, and tumor necrosis factor-alpha [TNF-α]). Gemcitabine significantly increased the plasma levels of IL-1α, IL-1β, and IL-17 compared to placebo (Figures 7A, B & D); DMAPT/gemcitabine treatment reduced these cytokine levels back to the placebo levels. DMAPT and DMAPT/gemcitabine significantly decreased the levels of IL-12p40, MCP-1 and TNF-α relative to placebo (Figures 7C, E & F). Although DMAPT and DMAPT/gemcitabine also reduced the levels of eotaxin and MIP-1β, the decreases were only significant for the combination (Figures 7G & H). Importantly, these eight cytokines are proinflammatory cytokines and known NF-κB target genes. Taken together, these results suggest that gemcitabine induces the expression of several pro-inflammatory NF-κB regulated cytokine genes. In contrast, treatment with DMAPT or the combination reduces the plasma levels of pro-inflammatory NF-κB-regulated cytokines, demonstrating NF-κB suppression by DMAPT.

## Discussion

In the present study, we evaluated the efficacy of the bioavailable NF-κB inhibitor DMAPT and gemcitabine using a genetically engineered and clinically relevant mouse model of pancreatic cancer. Much research supports a central role for NF-κB in inflammation and pancreatic tumorigenesis [8,9,11]. We have shown both *in vitro* and *in vivo* that pharmacological suppression of NF-κB by parthenolide or DMAPT has anti-cancer activity [15,16,33]. Gemcitabine, the current standard-of-care therapy for pancreatic cancer, has limited clinical benefit alone, and recent work has indicated that induction of NF-κB activity by gemcitabine may be involved in chemoresistance [9,17-20].

In this study, we report that gemcitabine and DMAPT/gemcitabine significantly increase median survival and decrease the incidence and multiplicity of pancreatic adenocarcinomas in *LSL-Kras*^*G12D/+*^*; LSL-Trp53*^*R172H*^*; Pdx-1-Cre* mice. Ki67, a cellular marker of proliferation, staining is reduced in both gemcitabine and the combination treatment groups, correlating with drug effects on tumor growth. The DMAPT/gemcitabine combination also significantly decreases tumor size as well as the incidence of metastasis to the liver. Although gemcitabine decreases tumor size compared to the placebo group, this effect is not significant. There is also no significant difference between the combination and gemcitabine alone groups with respect to tumor size. Furthermore, whereas liver metastasis in the combination group is significantly reduced compared to gemcitabine, the overall incidence of metastasis is not significantly different between the two groups due to metastasis occurring in the lung. Thus, it is not surprising that despite some additional anti-tumor effects of the combination, no further impact on survival is observed in our study when compared to gemcitabine treatment alone. Clinically, such differences, for example in tumor size, may be beneficial and translate to improved quality of life for the patient, without prolonging survival. Many variables (drug bioavailability and dose, treatment plan, specific mutations present etc.) can influence the effects of the drugs on tumor response *in vivo*. Thus, further optimization of the agents, experimental design, or animal model may be required to observe differential effects on survival.

No significant difference in premalignant PanIN formation is apparent between the treatment groups. This is likely due to the late stage of disease at the time of sacrifice and development of large pancreatic adenocarcinomas especially in the placebo and DMAPT treatment groups since in many cases, little or no histologically normal or premalignant pancreas was present. Further analysis specifically of pancreata lacking primary tumors reveals the presence of mainly normal ducts or PanIN-1 lesions, with only a few PanIN-2 and −3 lesions. This suggests that not only pancreatic tumors but also high grade pancreatic lesions are prevented in these animals. It will be of interest to further characterize the profiles of these “responders”.

We also report that gemcitabine treatment significantly increases the levels of the inflammatory cytokines IL-1α, IL-1β, and IL-17 in mouse plasma. The expression of these three genes is regulated by NF-κB, so this effect is consistent with gemcitabine-induced NF-κB activation that has been previously reported [34,35]. Treatment with DMAPT/gemcitabine reduces the levels of these cytokines to that of DMAPT alone. Furthermore, DMAPT and/or the combination DMAPT/gemcitabine significantly decrease the levels of the inflammatory cytokines IL-12p40, MCP-1, MIP-1β, eotaxin and TNF-α, all of which are NF-κB target genes [34]. Thus, the effect of DMAPT and DMAPT/gemcitabine on the expression of these cytokines suggests systemic suppression of the target NF-κB in these treatment groups. Target suppression was also demonstrated in our recent study with *LSL-Kras*^*G12D*^*; Pdx-1-Cre* genetically engineered mice in which all mice were treated for 3 months and then sacrificed 3 hours following the last drug treatment; specifically, combination DMAPT treatment decreased NF-κB expression in the pancreatic lesions [22]. Thus, at the 40 mg/kg dose, DMAPT consistently inhibits its target in models of either established or spontaneous tumors [15,16].

We previously showed that the percentage of normal pancreatic ducts was significantly increased by the combination of DMAPT/gemcitabine compared to placebo in the *LSL-Kras*^*G12D*^*; Pdx-1-Cre* mouse model; additionally, the percentage of mouse pancreatic intraepithelial neoplasia-2 (mPanIN-2) lesions was significantly decreased by DMAPT/gemcitabine [22]. This delay in PanIN progression in the *LSL-Kras*^*G12D*^*; Pdx-1-Cre* mice may in part explain the ability of the combination DMAPT/gemcitabine to attenuate pancreatic tumorigenesis in the present study. Similarly, Fendrich et al. recently showed that treatment with the angiotensin-I-converting enzyme inhibitor enalapril or aspirin, that targets NF-κB, delays PanIN progression in *LSL-Kras*^*G12D*^*; Pdx-1-Cre* mice and decreases pancreatic cancer development in the *LSL-Kras*^*G12D/+*^*; LSL-Trp53*^*R172H*^*; Pdx-1-Cre* mouse [36]. The combination of enalapril and asprin was not more effective than the single agents.

It has been previously reported by Olive et al. that gemcitabine therapy alone is ineffective in the *LSL-Kras*^*G12D/+*^*; LSL-Trp53*^*R172H*^*; Pdx-1-Cre* mouse model [37]. In contrast, gemcitabine alone in our study decreases tumor incidence in *LSL-Kras*^*G12D/+*^*; LSL-Trp53*^*R172H*^*; Pdx-1-Cre* mice. In our study, gemcitabine (50 mg/kg, twice weekly) was administered beginning at 1 month of age until the time of sacrifice/death. In Olive’s study, mice bearing tumors ~5-10 mm in diameter were identified and enrolled for gemcitabine treatment (100 mg/kg, Q3Dx4 or every third day for 4 cycles). In the latter study, gemcitabine was given to tumor-bearing mice for a much shorter time (9 days total); in contrast, for our study, gemcitabine was administered prior to any palpable tumor formation (tumors ~5-10 mm in diameter can be detected by palpation) at 1 month of age until ill health necessitated sacrifice or death occurred (~6 months of treatment). The earlier intervention as well as longer length of treatment may account for the difference in response between these two studies. Thus, based on these findings, gemcitabine administered earlier may have some ability to alter the development of pancreatic cancer. We speculate this may be an effect of delaying PanIN progression and/or possibly improved drug delivery prior to full stromal maturity of the developing adenocarcinomas. Gemcitabine has relatively low toxicity in humans, and at the comparably lower doses used in this study, there was no measurable toxicity in this model. Thus, the use of low dose gemcitabine at the time of diagnosis or in high risk groups may have benefit and merits further investigation.

## Conclusions

Although survival statistics for many cancers have improved in recent years, pancreatic cancer remains one of the deadliest diseases with a very poor survival rate due to the lack of early detection and absence of effective treatments. Patients at increased risk of pancreatic cancer include those with inheritable risk factors, patients presenting with premalignant pancreatic lesions, and individuals with multiple epidemiologic risk factors, including smoking, diabetes mellitus and obesity [38]. Identifying and monitoring such patients may improve their clinical outcome by allowing earlier surgical intervention or intervention with agents that can delay or prevent the progression of disease. Once pancreatic cancer develops, however, curative treatments are limited. Thus, novel and effective treatment strategies remain a critical area of research. In this study, gemcitabine alone and/or the combination of DMAPT and gemcitabine significantly increase median survival as well as decrease tumor size, the incidence and multiplicity of pancreatic tumors, and metastasis to the liver in *LSL-Kras*^*G12D/+*^*; LSL-Trp53*^*R172H*^*; Pdx-1-Cre* mice. The preclinical evidence presented here supports further evaluation of either gemcitabine alone or in combination with DMAPT or related agents as intervention to delay the progression of pancreatic cancer.

## Competing interests

P.A.C. has a financial interest in Leuchemix, Inc. All others do not have any competing interest.

## Authors’ contributions

MYS designed the study, participated in the animal treatment, performed analyses, and drafted the manuscript. HW bred and genotyped the animals and participated in animal treatment. KS performed the imaging analysis and helped revise the manuscript. NA determined histopathology of the specimens. PC synthesized the DMAPT and helped revise the manuscript. CMS participated in study design and helped draft the manuscript. All authors read and approved the final manuscript.

## Pre-publication history

The pre-publication history for this paper can be accessed here:

http://www.biomedcentral.com/1471-2407/13/194/prepub

## Supplementary Material

Additional file 1: Figure S1 Confirmation of Cre-mediated recombination in the pancreas. Cre-mediated recombination in the pancreas was confirmed by performing PCR to detect the single Lox P site in nontumor-bearing mice (lanes 1-14). A DNA ladder (L) as well as positive (+) and negative (-) PCR controls were run in parallel. Bands corresponding to the single Lox P site and wild-type (WT) are indicated. Click here for file
