# Supplementary figures and images for: Dimethylaminoparthenolide and gemcitabine: a survival study using a genetically engineered mouse model of pancreatic cancer
Source: BMC Cancer. 2013 Apr 17;13:194. doi: 10.1186/1471-2407-13-194 (PMC3672012; doi:10.1186/1471-2407-13-194)

## Slide 1
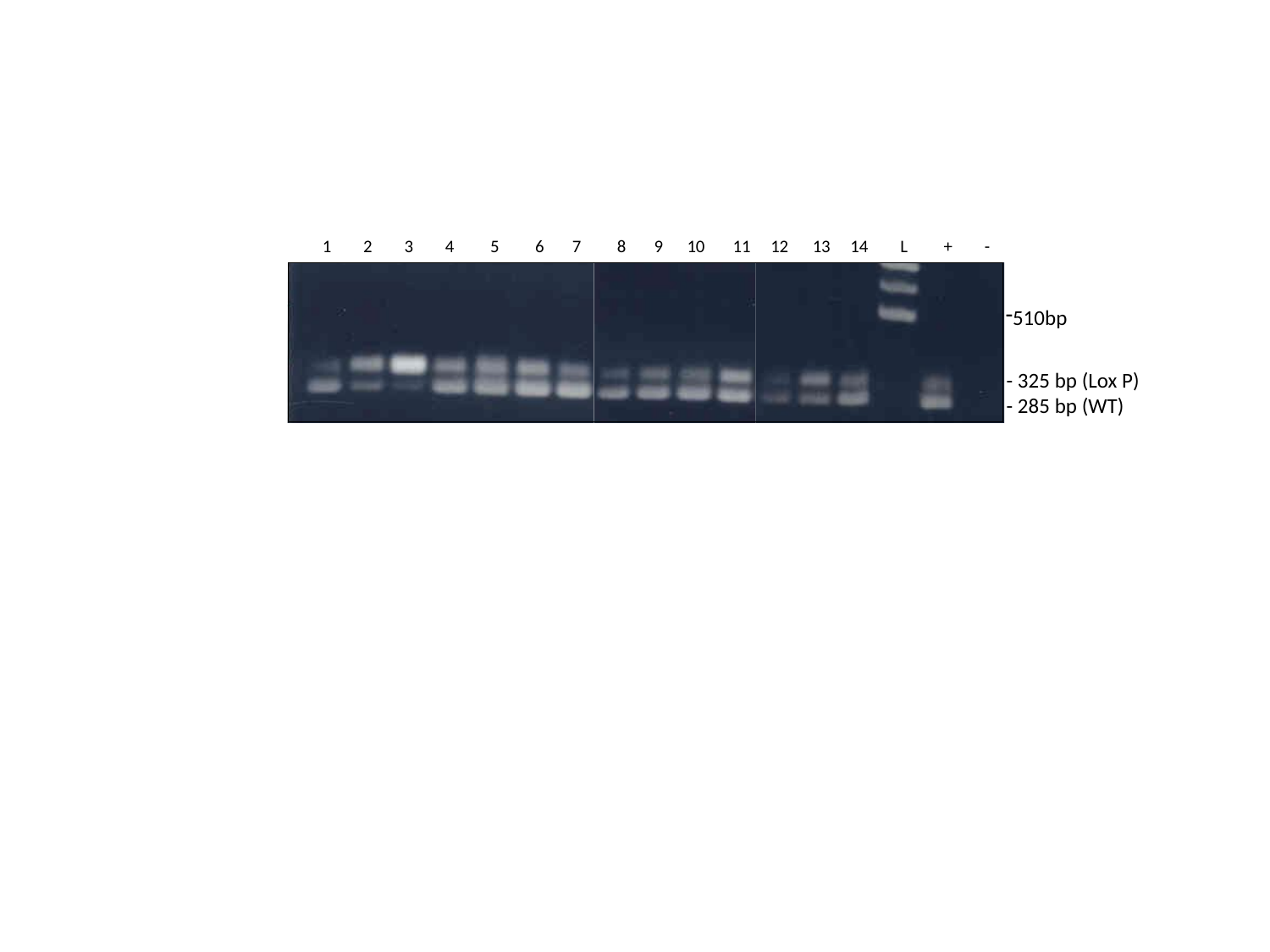

1 2 3 4 5 6 7 8 9 10 11 12 13 14 L + -
510bp
- 325 bp (Lox P)
- 285 bp (WT)

Supplement: Additional file 1: Figure S1 — Confirmation of Cre-mediated recombination in the pancreas. Cre-mediated recombination in the pancreas was confirmed by performing PCR to detect the single Lox P site in nontumor-bearing mice (lanes 1-14). A DNA ladder (L) as well as positive (+) and negative (-) PCR controls were run in parallel. Bands corresponding to the single Lox P site and wild-type (WT) are indicated. [file 1471-2407-13-194-S1.pptx]
